# Denial of reproductive potential: a predictor of unperceived pregnancy in an Austrian neonaticide sample

**DOI:** 10.1007/s00737-024-01481-x

**Published:** 2024-06-19

**Authors:** Claudia M. Klier, Bozic Ina, Yvonne Kuipers, Sabine Amon

**Affiliations:** 1 Department of Pediatrics and Adolescent Medicine, Medical University of Vienna, Vienna, Austria; 2 Department of Psychiatry and Psychotherapeutic Medicine, Medical University of Vienna, Vienna, Austria; 3 School of Health and Social Care, Edinburgh Napier University, Edinburgh, Scotland, United Kingdom; 4 Barmherzige Brüder Hospital, Vienna, Austria

**Keywords:** Unperceived pregnancy, Neonaticide, Denial of fertility, Denial of pregnancy, Perinatal psychiatry, Contraception, Unassisted birth

## Abstract

**Purpose:**

This study aims to describe the phenomenon of unperceived pregnancy followed by neonaticide with a focus on the lack of awareness of reproductive potential in an Austrian sample.

**Methods:**

An explorative comparative study of neonaticide cases with single and repeat perpetrators was conducted using nationwide register-based data from 1995 to 2017. A total number of 55 cases out of 66 were included in the analysis. A standardized coding sheet was used and calculations were performed.

**Results:**

48 women gave birth to 101 children, of which 55 were killed, 23 children lived out of home care and 23 lived with the perpetrator We found a higher fertility rate in both neonaticide perpetrators in the single (1,9) and the repeat group (4,25) in comparison to the general population (1,4). The use of contraception was only 31% among neonaticide perpetrators, deviating substantially from the general Austrian population age group (16-29yrs) which used contraception in 91%. The neonaticide perpetrators used an effective contraception method (pearl-index < 4) in only 2%, whereas 20% of the general population did so. The number of unperceived pregnancies was high in both groups (50/55) 91%.

**Conclusion:**

Future case reports and forensic evaluations should take reproductive behavior into account, as it may offer valuable insights into the events leading up to neonaticide. Our findings suggest that denial of reproductive potential often precedes unperceived pregnancies. In the Austrian cohort, women who experienced unperceived pregnancies resulting in unassisted births and subsequent neonaticide showed a low prevalence of contraceptive use. This is particularly noteworthy given that the primary motive for neonaticide is unwanted pregnancy.

## Introduction

Historically the phenomenon of women who claimed to have not been aware of a pregnancy before giving birth has been termed denied or concealed pregnancy. The terminology is often used interchangeably as researchers debate whether the pregnant women have any degree of awareness and to what degree they are actively concealing the pregnancy (Murphy-Tighe and Lalor 2019). Denial can be regarded as a psychological mechanism of defense or protection against the anticipated negative consequences of a pregnancy and the avoidance of the memory of traumatizing experiences (Bonnet 2021). Similarly, concealment can be understood to be a fear-induced coping mechanism that results in avoidance strategies, such as wearing loose-fitting clothing to avoid confrontations (Murphy-Tighe and Lalor 2019). Due to this overlap, we have adopted the terminology of unperceived pregnancy in accordance with Şar et al. (2017) and Barnes (2022), who stress that these women do not perceive the pregnancy-related symptoms or misattribute them to other bodily sensations.

Unperceived pregnancy is more common than thought and was reported at a rate of one in every 300 pregnancies up to the 20th week of gestation (Simermann et al. 2018) or one in every 475 pregnancies (Wessel 2002). The number of completely unexpected, abrupt births of a viable newborn (intrapartum diagnosis of the pregnancy) occurs at a rate of one in every 2455 pregnancies (Wessel et al. 2002). Unperceived pregnancy has been a subject of interest in the forensic research community as early as the 1960s (Gerchow 1964), as it is the most important risk factor for neonaticide (Amon et al. 2012). Neonaticide is the criminal act of ending the life of a newborn within the first 24 h after birth, usually committed by the biological mother (Resnick 1970). Physical symptoms of pregnancy, for example, weight gain and amenorrhea going unnoticed by both the women and their social environment might be difficult to comprehend. However, a large population-based study found that in 38% of the cases, the unperceived pregnancy was also missed by doctors who were treating symptoms that were retrospectively well explained by the pregnancy (Beier et al. 2006). Wessel (2002) compared reports from German women who had experienced an unperceived pregnancy with reports from neonaticide perpetrators from Gerchow (1964) and found no differences regarding concealment and denial, which suggests that these processes are complementary, rather than mutually exclusive. It is noteworthy, that Murphy-Tighe and Lalor (2019) documented conscious awareness of the pregnancies in women who committed neonaticide, however, this research was conducted in the form of retrospective interviews.

There are common characteristics found in neonaticide perpetrators that could point to predictive risk factors (Amon et al. 2012; Friedman et al. 2007). For example, Wille et al. (2003) found that inadequate conflict-resolution skills, conflict-avoidance tendencies, high harmony-seeking trait, and low self-esteem were contributing factors. Trauma is known to be underreported generally and in neonaticide cases specifically because the focus is often placed on personality factors. However, a history of trauma, abuse and neglect in childhood or current life is reported by up to 48% of neonaticide perpetrators (Bonnet 1993; Klier et al. 2019). To further understand these phenomena, a distinction should be made between single perpetrators, who commit one offense and repeat perpetrators, who committed neonaticide multiple times (Klier et al. 2019).

There is increasing evidence for the role of trauma in the psychopathology behind neonaticide which was precipitated by an unperceived pregnancy (Barnes 2022). The course of an unperceived pregnancy can be interpreted as a dissociative disorder and a fearful, life-defining, traumatic experience, likely to be followed by a traumatic birth, potentially leading to detrimental outcomes for the newborn and their mothers – a traumatic experience that does not necessarily end when the baby has been born (Barnes 2022; Leinweber et al. 2022; Murphy-Tighe and Lalor 2019).

Our research is concerned with the reproductive behaviour of neonaticide perpetrators, specifically the deviations from the national average in terms of rate of births and choice of contraceptive method. Struye et al. (2013) state the hypothesis that women who experience an unperceived pregnancy that leads to the act of neonaticide exhibit denial regarding their reproductive potential. The term “awareness of reproductive potential” is best understood as the ability to make a link between sexual intercourse and the possibility of this act to lead to a pregnancy. The phenomenon is perplexing as these women do not want to become pregnant, but they very rarely use contraception (Struye et al. 2013). Struye et al. present a case study of a woman who had seven unintended pregnancies (three births followed by three anonymous adoptions following unperceived pregnancies and one neonaticide) in which the woman could not explain why she had not used contraception and stated that she was thinking each time it would not happen. Struye et al. (2013) point out the high fertility rate in this case. Twenty years prior to this publication, Bonnet (1993) interviewed 18 women who experienced an unperceived pregnancy of which 14 had given up their child for adoption and 4 had committed neonaticide. Bonnet concluded that the unperceived pregnancy was preceded by denial regarding the female sexuality and reproductive potential and that they did therefore not use contraception. The women reported neglect and sexual trauma during childhood, as well as, sexual violence or domestic violence in their adult relationships (Bonnet 1993).

In a recent study by Delong et al. (2022) women with unperceived pregnancy without traumatic history had a high rate of contraceptive usage (75%). Surprisingly, in 86% of cases, menstruation continued throughout the pregnancy. Delong et al. (2022) suggest that a positive family history and previous unperceived pregnancies are risk factors. This finding is of relevance regarding the analysis of differences and similarities in psychopathology and characteristics between single and repeat perpetrators of neonaticide (Klier et al. 2019).

Although the samples sizes in the aforementioned studies were small, the largest comprising of 71 women with unperceived pregnancies (Delong et al. 2022) and 28 women committing neonaticide (Amon et al. 2012), the findings suggest a possible connection between unperceived fertility, lack of contraception usage and unperceived pregnancy, which in turn is an established risk factor for neonaticide. Additional research is needed to validate this hypothesis using neonaticide register-based data. The motives of women who kill a newborn identified so far include unperceived pregnancy (Amon et al. 2012) and unwanted child (Putkonen et al. 2016). This study is conducted to elucidate the connection between an unwanted pregnancy and the use or non-use of contraception.

## Materials and methods

### Data collection

The present study used nationwide register-based comprehensive data collected in Austria from 1995 to 2017. According to the definition of neonaticide (Resnick 1970) we analysed cases of newborns who died within 24 h of birth. The Austrian Coroner Law requires the identification of a cause of death by autopsy in cases under the age of 18 (Hochmeister et al. 2007). We had access to coroner reports and court files which included police reports (interrogation of perpetrator, family, colleagues, neighbours, and analysis of crime scene), psychiatric evaluations, previous criminal records and verdicts. The exclusion criteria for this analysis were an unknown perpetrator and missing files.

All cases were analysed by one researcher (SA) by using a standardized coding sheet including 519 variables, which were chosen based on an extensive review of the literature (Amon et al. 2012; Putkonen et al. 2009; Putkonen et al. 2016). Most of the variables were dichotomous at a nominal level and involved demographic information of the perpetrator and the victim, social factors, motives, education, living situation, pregnancy awareness, contraception, psychiatric examinations and verdict, all entered into SPSS 21 for statistical analysis. 

The Pearl Index measures the effectiveness of a contraceptive method by calculating the number of pregnancies occurring per 100 women-years of exposure. It provides a standardized way to compare the failure rates of different contraceptive methods. This measure was used to compare the contraceptive behavior.

### Analysis

Descriptive statistics were used to compare variables between single and repeated perpetrators, including independent-test analysis and Chi-square. The characteristics of maternal age and parity were compared with the Austrian national data from the Statistic Austria Institute. All results were calculated by using the mean values of the study period. The data on contraception use by the Austrian population were extracted from a report published in 2019.

## Results

In Austria, 69 cases of neonaticide were recorded between 1995 and 2017 of which14 cases were excluded (6 cases due to an unknown perpetrator and 8 cases due to missing files). We analysed 55 cases of neonaticide, of which 44 cases (80%) were conducted by single perpetrators. The remaining 11 neonaticides (20%) were committed by 4 women, which we will refer to as the repeat perpetrators. (see Table 1).

**Table 1:** Characteristics Austrian cases/ perpetrators and the general population

	Single perpetrators *N* = 44	Repeat perpetrators *N* = 4	Population AUT ^1,2^
	Mean (SD)	Mean (SD)	
Number inhabitants	--	--	8,264,390
Age at delivery (years)*	25.6 (7.2)	30.8 (6.2)	29.6
Females in fertile age group (15–49 years)			2,072,943
Total number of children	84	17	80,518.48 (per year)
Number of abortions	3	0	no data^3^
Alive birth-rate per 1.000 inhabitants	--	--	9,76
Number of killed neonates/ newborn	44	11	307.09 (per year)
Neonatal mortality-rate per 1.000 births	--	--	3.79
Fertility-rate**	1.9 (1.24)	4.25 (0.5)	1.41

^1^Statistics Austria 2018

^2^Over the study period all measures were calculated as means and standard deviations

^3^No data available for Austria (Fiala et al. 2022)

*[T-Test: df = 53, *p* = 0.03]

**[T-Test: df = 46, *p* = 0.001]

A total of 101 children were born by the perpetrators, of the surviving 46 children, 23 lived out of home care and 23 lived with their mother. All 6 [Mean = 1.5, SD = 1.29] living children of the repeat perpetrators were in out-of-home care, whereas the single perpetrators had 23 live children of which 17 were not living with their mother [Mean = 0.52, SD = 0.98]. The difference was not significant [T-Test: df = 46, *p* = 0.68]. There were three recorded terminations of pregnancy, all undergone by different single perpetrators. The ages of the perpetrators were categorised into four age groups according to the grouping of the Austrian Statistic Institute. Table 2 shows that the four repeat perpetrators had their first child between the ages of 21 to 29 years, while the maternal age at first births in the single perpetrator group varied. The difference was not significant [Chi-square = 7.273, df = 3, *N* = 48, *p* = 0.064].

**Table 2:** Age of first biological offspring

	Single perpetrators *N* = 44	Repeat perpetrators *N* = 4	Population AUT^1^ annual birth rate
< 15 years	0	0	< 1%
15–20 years	26 (59%)	0	3%
21–29 years	14 (32%)	4 (100%)	45%
30 years or older	4 (9%)	0	52%

^1^Statistic Austria 4: 2007–2017: annual birth rate clustered by age of mothers

### Psychiatric diagnosis

More than half of the offenders had no diagnosis at the time of the crime. The second largest group comprised perpetrators with personality disorders, accounting for 18.8%, followed by mood disorders, which affected 10.4% of the women. Psychotic disorders and learning disabilities were each diagnosed in only 3 women (6.3% for each group). In the repeat offender group, 3 out of 4 had a personality disorder. Data is missing for the remainder.

### Contraception and termination of pregnancy

Only 31% of all perpetrators used any contraception method. Among the repeat perpetrators in 4 of the 11 cases (36%) (5 cases unknown) the partner was not aware of the contraception status of their partner. Among the single perpetrators, this occurred in 3 out of 44 cases (7%) (10 cases unknown). The difference showed that repeat perpetrators obscured the contraception status significantly more often to their partner compared to single perpetrators [Chi-square = 11.819, df = 1, *N* = 40, *p* = 0.001]. The male partners of repeat perpetrators showed significantly more often an interest in considering the use of contraception, being 9 of 11 cases (82%) (2 cases unknown) compared to male partners of single perpetrators, being 10 out of 44 cases (23%) (11 cases unknown) [Chi-square = 13.866, df = 1, *N* = 42, *p* = 0.000]. In total 34% of male partners had an interest in contraception according to police records.

In Austria, 77% of all women in the fertile age group (16–49 years) use any type of contraception, varying in methods. In the age group of women aged 16 to 29 years (comprising 91% of perpetrators) even 91% of the general population used contraception (see Table 3).

**Table 3:** Contraception

	Single perpetrators *N* = 44	Repeat perpetrators *N* = 11	All perpetrators *N* = 55	Population AUT
Contraception prevalence total	13 (35%)	4 (44%)	17 (31%)	91%^1^
Pearl index < 4 (injectables, implant, sterilization, IUS)	1 (2%)	0	1 (2%)	20%^2^
Pearl index 4–10 (pill, intravaginal ring, contraceptive patch)	7 (16%)	0	7 (13%)	34%^2^
Pearl index 10–20 (condom)	4 (9%)	4 (36%)	8 (15%)	38%^2^
Pearl index > 20 (withdrawal)	1 (2%)	0	1 (2%)	11%^2^
Not using contraception	24 (55%)	5 (45%)	29 (53%)	26%^2^
Unknown contraception status	7 (16%)	2 (18%)	9 (16%)	--
Number of unperceived pregnancies	39 (87%)	11 (100%)	50 (91%)	1/475 (2‰)^3^

^1^ Contraception report Austria 2012: The percentage of women aged 16–29 years, married or partnership, who are currently using, or whose sexual partner is using, at least one method of contraception, regardless of the method used

^2^ Contraception report Austria 2012

^3^ Wessel et al. 2002

The Austrian contraception report differentiated between the methods using the Pearl Index showing that only 15% of the perpetrators used a safe method of contraception (Pearl Index < 4–10), whereas 54% of the general population did so.

### Unperceived pregnancy

In 50 out of 55 neonaticide cases (91%), the women reported signs of an unperceived pregnancy prior to neonaticide. All 4 repeat perpetrators reported signs of an unperceived pregnancies for all 11 neonaticides that were committed. The single perpetrators’ pregnancies were unperceived in 39 of 44 cases (89%).

## Discussion

To our knowledge, this is the first of published data on contraceptive use and fertility rate including a comparison of a single to a repeat perpetrator group of women in a large register-based neonaticide sample. The background of this undertaking were case reports of women who killed a newborn, who had a high fertility rate, unwanted pregnancies and nevertheless did not use contraception. The overall contraception prevalence in the Austrian population is 77%, in the age group of women 16 to 29 years, this percentage goes up to 91% which makes the difference to the neonaticide group even more striking. The reasons for ignoring the possibility of pregnancy without contraception were analyzed by Fiala, an Austrian reproductive scientist (Fiala 2019, Austrian contraception report), who described rare sexual intercourse, supposed infertility, the firm conviction that nothing will happen, more pleasure without contraception, and disagreement of hormonal contraception as motives. Fiala (2019) concluded that a lack of knowledge regarding the real frequency of possible pregnancies in a lifespan is responsible. The actual number of these is 12 to 15 during the 35-year average of female fertility. One-quarter of reproductive women in the report believed that the possibility of lifespan pregnancies was 0–3, a number which increased with higher education level and age but was still far from the correct number. In the perpetrator group, there must be additional factors that lead to such dysfunctional reproductive behavior resulting in this high fertility rate. Notably a case report (Struye et al. 2013) describes a mother of 6 (3 out of home care) who becomes pregnant with her 7th pregnancy resulting in neonaticide and who never used any contraception.

Neonaticide perpetrators showed some differences compared to the general Austrian population in several other aspects of reproductive parameters. Neonaticide perpetrators showed even less awareness concerning their potential to conceive. More than half of them did not use any kind of contraception and only 2% used a method with a Pearl Index of less than 4 (for instance injectables, IUD) in contrast to the general population who used these in 20%. The non-awareness of pregnancy in combination with the male partner’s lack of interest in contraception and obscuring the fact that they did not use contraception offers a theory about the quality of the relationship and the responsibility of male partners. In our sample, 34% percent of the male partners communicated (police records) an interest in contraception, whereas 36% of the perpetrators obscured the contraception status. A limiting factor of such analyses is that it cannot be verified. Our data showed that contraception status was unknown in nearly every fifth case.

It could be hypothesized, that there was no real mature interest in contraception by the male partners and that they did not take responsibility for their contraception possibilities (like vasectomy or condoms). The explanation that a third of the perpetrators were vague about that, could be interpreted as immature or dependent personality factors within that relationship. Regardless of the responsibility none of the partners integrate a mature discussion of reproduction in their relationship, although a third of them already lived with children, a third with children living out of home and a third were single mothers.

The single perpetrators showed more variations in their motives for unperceived pregnancies, half of them had no explanation or did not know what to do in that situation. The factor of young age seems like a potential indicator (Amon et al. 2012), but more questionable was also the quality of the relationships. It can be hypothesized that the sample of neonaticide perpetrators exhibited limited knowledge of contraception and inadequate cognitive processing regarding birth control. The awareness of the pregnancy by their social surroundings and their partner’s emotional involvement in the perpetrator’s life was astonishingly low.

The high percentage of women not using any contraception, a high fertility rate together with the high percentage (90%) of unperceived pregnancies in neonaticide offenders, might propose that other factors than contraception knowledge, obscuring of contraception status or use of unsafe contraception need to be taken into account.

From previous work, we know that 48% of perpetrators have had a traumatic experience in their childhood or actual life (Klier et al. 2019), but trauma is frequently underreported. In the present study this number was lower (35%). Bonnet who used depth-psychological interviews with women who either killed their newborns or had them delivered anonymously and given up for adoption, found trauma, abuse and neglect in nearly all the cases (Bonnet 1993). The course of an unperceived pregnancy can be interpreted as a dissociative disorder (Barnes 2022) and is followed by a likely traumatic birth (Leinweber et al. 2022) leading to detrimental outcomes for the babies and their mothers. The whole issue of trauma in the history of these women could only be assessed by interviews by an experienced clinician rather than the evaluation of the files from the court, police and forensic expertise, which might have led to the lack of information in our sample on this issue. The gap in reports of a traumatic birth experience was described recently in principle-based concept analyses, which could only include 6 papers offering descriptions of women’s experience of the delivery after an unperceived pregnancy (Kuipers et al. 2024). The results from the psychiatric diagnoses show that more than half of the subjects did not have any psychiatric diagnosis. Only a few had personality or affective disorders. Notably, there were no diagnoses of posttraumatic stress disorder in this sample, which might be due to underreporting.

## Conclusions

This paper describes the low contraception use and a high fertility rate in women with unperceived pregnancies, followed by neonaticides. This can hardly be explained by a general lack of basic knowledge regarding reproductive biology and lack of sexual education. The population of neonaticide offenders differs substantially from the general population when it comes to the use of safe contraceptive methods, which has to be explained differently. We assume that dissociative symptoms occur frequently in this population and not only the pregnancy and birth are affected by them. It can be suspected that this process of denial starts well before the actual pregnancy and leads to a denial of the acknowledgment of their reproductive potential. This pathway has to be elucidated more rigorously by forensic experts and experts in the field of perinatal psychiatry delivering a “reproductive roadmap” for each case. This means that all reproductive events including the psychological reaction of self and others to menarche, first sexual encounters, choice of partners, possible sexual abuse, neglect, intimate partner violence, abortions, previous unperceived pregnancies, previous miscarriages, fear of obstetricians/doctors, contraception knowledge and use, cultural and familial factors around the topic of sexuality and their significance to the course of events have to be collected comprehensively. Only this can ensure that the context of the unperceived pregnancy with sometimes dramatic outcomes can be understood on an individual level.

## References

[CR1] Amon S, Putkonen H, Weizmann-Henelius G, Almiron MP, Formann AK, Voracek M, Eronen M, Yourstone J, Friedrich M, Klier CM (2012) Potential predictors in neonaticide: the impact of the circumstances of pregnancy. Archives Women’s Mental Health 15(3):167–174. 10.1007/s00737-012-0268-0 10.1007/s00737-012-0268-0 22426944

[CR2] Barnes DL (2022) Towards a new understanding of pregnancy denial: the misunderstood dissociative disorder. Archives Women’s Mental Health 25(1):51–59. 10.1007/s00737-021-01176-7 10.1007/s00737-021-01176-7 34392438

[CR3] Beier KM, Wille R, Wessel J (2006) Denial of pregnancy as a reproductive dysfunction: a proposal for international classification systems. J Psychosom Res 61(5):723–730. 10.1016/j.jpsychores.2005.11.002 17084153 10.1016/j.jpsychores.2005.11.002

[CR4] Bonnet C (1993) Adoption at birth: Prevention against abandonment or neonaticide. Child Abuse Negl 17(4):501–513. 10.1016/0145-2134(93)90025-z 8402253 10.1016/0145-2134(93)90025-z

[CR5] Bonnet C (2021) Description Clinique et accompagnement pluridisciplinaire du déni de grossesse. Sages-Femmes 20(2):10–15. 10.1016/j.sagf.2021.01.003

[CR6] Delong H, Eutrope J, Thierry A, Sutter-Dallay A, Vulliez L, Gubler V, Saad Saint‐Gilles S, Tessier E, Le Foll J, Viaux S, Apter G, Danion A, Auer J, Rolland A (2022) Pregnancy denial: a complex symptom with life context as a trigger? A prospective case–control study*. BJOG 129(3):485–492. 10.1111/1471-0528.16853 34324258 10.1111/1471-0528.16853 PMC9291172

[CR7] Fiala C, Agostini A, Bombas T, Lertxundi R, Lubusky M, Parachini M, Gemzell-Danielsson K (2022) Abortion: legislation and statistics in Europe. Eur J Contracept Reproductive Health Care: Official J Eur Soc Contracept 27(4):345–352. 10.1080/13625187.2022.2057469 10.1080/13625187.2022.2057469 35420048

[CR8] Friedman SH, Heneghan A, Rosenthal M (2007) Characteristics of women who deny or conceal pregnancy. Psychosomatics 48(2):117–122. 10.1176/appi.psy.48.2.117 17329604 10.1176/appi.psy.48.2.117

[CR9] Gerchow J (1964) Schwangerschaft Und Geburt unter medizinisch-forensischen Aspekten Der Kindestötung. Monatsschrift für Kriminol Und Strafrechtsreform 47(6):233–240. 10.1515/mks-1964-470601

[CR10] Hochmeister M, Grassberger M, Stimpfl T (2007) Forensische Medizin für Studium Und Praxis. Maudrich, Wien

[CR12] Klier CM, Amon S, Putkonen H, Arias F, P., Weizmann-Henelius G (2019) Repeated neonaticide: differences and similarities to single neonaticide events. Archives Women’s Mental Health 22(1):159–164. 10.1007/s00737-018-0850-1 10.1007/s00737-018-0850-1 PMC6373254 29796966

[CR11] Kuipers Y, Thomson G, Škodová Z, Bozic I, Lísa Sigurðardóttir V, Goberna-Tricas J, Zurera A, Neves DM, Barata C, Klier C (2024) A multidisciplinary evaluation, exploration, and advancement of the concept of a traumatic birth experience. Women Birth: J Australian Coll Midwives 37(1):51–62. 10.1016/j.wombi.2023.08.004 10.1016/j.wombi.2023.08.004 37658018

[CR13] Leinweber J, Fontein-Kuipers Y, Thomson G, Karlsdottir S I, Nilsson C, Ekström-Bergström A, Olza I, Hadjigeorgiou E, Stramrood C (2022) Developing a woman-centered, inclusive definition of traumatic childbirth experiences: a discussion paper. Birth (Berkeley Calif) 49(4):687–696. 10.1111/birt.12634 35403241 10.1111/birt.12634

[CR14] Murphy-Tighe S, Lalor JG (2019) Regaining agency and autonomy: a grounded typology of concealed pregnancy. J Adv Nurs 75(3):603–615. 10.1111/jan.13875 30307061 10.1111/jan.13875

[CR15] Putkonen H, Amon S, Almiron MP, Cederwall JY, Eronen M, Klier C, Kjelsberg E, Weizmann-Henelius G (2009) Filicide in Austria and Finland—A register-based study on all filicide cases in Austria and Finland 1995–2005. BMC Psychiatry 9:74. 10.1186/1471-244X-9-74 19930581 10.1186/1471-244X-9-74 PMC2784763

[CR16] Putkonen H, Amon S, Weizmann-Henelius G, Panakoski M, Eronen M, Klier CM (2016) Classifying Filicide. Int J Forensic Mental Health 0:0,1–13. 10.1080/14999013.2016.1152616

[CR17] Resnick G, P. J (1970) Murder of the newborn: a psychiatric review of neonaticide. Am J Psychiatry 126(10):1414–1420. 10.1176/ajp.126.10.1414 5434623 10.1176/ajp.126.10.1414

[CR18] Şar V, Aydın N, van der Hart O, Steven Frankel A, Şar M, Omay O (2017) Acute dissociative reaction to spontaneous delivery in a case of total denial of pregnancy: diagnostic and forensic aspects. J Trauma Dissociation: Official J Int Soc Study Dissociation (ISSD) 18(5):710–719. 10.1080/15299732.2016.1267685 10.1080/15299732.2016.1267685 27997287

[CR19] Simermann M, Rothenburger S, Auburtin B, Hascoët J-M (2018) Outcome of children born after pregnancy denial. Archives De Pediatrie: Organe Officiel De La Societe Francaise De Pediatr 25(3):219–222. 10.1016/j.arcped.2018.01.004 10.1016/j.arcped.2018.01.004 29523380

[CR20] Statistik Austria: Demographisches Jahrbuch 2017 (2018) https://www.statistik.at/fileadmin/publications/Demographisches_Jahrbuch_2017.pdf. Accessed 13.2.2024

[CR21] Struye A, Zdanowicz N, Ibrahim C, Reynaert C (2013) Can denial of pregnancy be a denial of fertility? A case discussion. Psychiatria Danubina 25(Suppl 2):113–117 23995158

[CR22] Contraception report (Verhütungsreport) (2012) https://verhuetungsreport.at/. Accessed 13.2.2024

[CR23] Contraception report (Verhütungsreport) (2019) https://verhuetungsreport.at/. Accessed 13.2.2024

[CR24] Wessel J (2002) Denial of pregnancy: Population based study. BMJ (Clinical Research Ed. 324(7335):458. 10.1136/bmj.324.7335.458 10.1136/bmj.324.7335.458 PMC65667 11859048

[CR25] Wessel J, Endrikat J, Buscher U (2002) Frequency of denial of pregnancy: results and epidemiological significance of a 1-year prospective study in Berlin. Acta Obstet Gynecol Scand 81(11):1021–1027. 10.1034/j.1600-0412.2002.811105.x 12421169 10.1034/j.1600-0412.2002.811105.x

[CR26] Wille R, Steckeler U, Wessel J (2003) Zur Kollusion Zwischen Juristen und medizinischen Gutachtern in Strafprozessen Wegen Tötung Unter Der Geburt, vol 10. Sexuologie - Urban & Fischer, pp 61–77

